# Supplementary material for: Functional neural networks stratify Parkinson's disease patients across the spectrum of cognitive impairment
Source: Brain Behav. 2024 Jan 24;14(1):e3395. doi: 10.1002/brb3.3395 (PMC10808882; doi:10.1002/brb3.3395)
Supplement: Supplementary file 1 — Post hoc findings. [file BRB3-14-e3395-s002.docx]

Supplementary file 1-post-hoc findings

|  |  | | | **Mean Difference** | **Std. Error** | **Confidence Interval** | | **p value** |
| --- | --- | --- | --- | --- | --- | --- | --- | --- |
|  |  |  |  |  |  | **Lower Bound** | **Upper Bound** |  |
| **Attention** | **Digit span forward** | HC (n=26) | PD-NC (n=25) | -1.472 | 0.383 | -2.504 | -0.439 | **0.001*** |
|  |  |  | PD-MCI (n=32) | 0.346 | 0.357 | -0.616 | 1.308 | 1.000 |
|  |  |  | PDD (n=31) | 1.274 | 0.360 | 0.304 | 2.245 | **0.004*** |
|  |  | PD-NC (n=25) | HC (n=26) | 1.472 | 0.383 | 0.439 | 2.504 | **0.001*** |
|  |  |  | PD-MCI (n=32) | 1.818 | 0.374 | 0.810 | 2.825 | ***< 0.001**** |
|  |  |  | PDD (n=31) | 2.746 | 0.377 | 1.731 | 3.761 | ***< 0.001**** |
|  |  | PD-MCI (n=32) | HC (n=26) | -0.346 | 0.357 | -1.308 | 0.616 | 1.000 |
|  |  |  | PD-NC (n=25) | -1.818 | 0.374 | -2.825 | -0.810 | ***< 0.001**** |
|  |  |  | PDD (n=31) | 0.928 | 0.350 | -0.015 | 1.872 | 0.056 |
|  |  | PDD (n=31) | HC (n=26) | -1.274 | 0.360 | -2.245 | -0.304 | **0.004*** |
|  |  |  | PD-NC (n=25) | -2.746 | 0.377 | -3.761 | -1.731 | ***< 0.001**** |
|  |  |  | PD-MCI (n=32) | -0.928 | 0.350 | -1.872 | 0.015 | 0.056 |
|  | **Digit span backward** | HC (n=26) | PD-NC (n=25) | -0.111 | 0.312 | -0.952 | 0.728 | 1.000 |
|  |  |  | PD-MCI (n=32) | 0.580 | 0.291 | -0.203 | 1.364 | 0.293 |
|  |  |  | PDD (n=31) | 1.615 | 0.293 | 0.824 | 2.405 | ***< 0.001**** |
|  |  | PD-NC (n=25) | HC (n=26) | 0.111 | 0.312 | -0.728 | 0.952 | 1.000 |
|  |  |  | PD-MCI (n=32) | 0.692 | 0.304 | -0.127 | 1.513 | 0.151 |
|  |  |  | PDD (n=31) | 1.727 | 0.307 | 0.900 | 2.554 | ***< 0.001**** |
|  |  | PD-MCI (n=32) | HC (n=26) | -0.580 | 0.291 | -1.364 | 0.203 | 0.293 |
|  |  |  | PD-NC (n=25) | -0.692 | 0.304 | -1.513 | 0.127 | 0.151 |
|  |  |  | PDD (n=31) | 1.034 | 0.285 | 0.265 | 1.803 | **0.003*** |
|  |  | PDD (n=31) | HC (n=26) | -1.615 | 0.293 | -2.405 | -0.824 | ***< 0.001**** |
|  |  |  | PD-NC (n=25) | -1.727 | 0.307 | -2.554 | -0.900 | ***< 0.001**** |
|  |  |  | PD-MCI (n=32) | -1.034 | 0.285 | -1.803 | -0.265 | **0.003*** |
| **Executive functions** | **Stroop spontaneous correction** | HC (n=26) | PD-NC (n=25) | -0.160 | 0.853 | -2.469 | 2.147 | 1.000 |
|  |  |  | PD-MCI (n=32) | -3.032 | 0.833 | -5.288 | -0.776 | **0.003*** |
|  |  |  | PDD (n=31) | -1.433 | 1.059 | -4.300 | 1.432 | 1.000 |
|  |  | PD-NC (n=25) | HC (n=26) | 0.160 | 0.853 | -2.147 | 2.469 | 1.000 |
|  |  |  | PD-MCI (n=32) | -2.871 | 0.869 | -5.223 | -0.518 | **0.009*** |
|  |  |  | PDD (n=31) | -1.272 | 1.087 | -4.215 | 1.670 | 1.000 |
|  |  | PD-MCI (n=32) | HC (n=26) | 3.032 | 0.833 | 0.776 | 5.288 | **0.003*** |
|  |  |  | PD-NC (n=25) | 2.871 | 0.869 | 0.518 | 5.223 | **0.009*** |
|  |  |  | PDD (n=31) | 1.598 | 1.072 | -1.303 | 4.500 | 0.840 |
|  |  | PDD (n=31) | HC (n=26) | 1.433 | 1.059 | -1.432 | 4.300 | 1.000 |
|  |  |  | PD-NC (n=25) | 1.272 | 1.087 | -1.670 | 4.215 | 1.000 |
|  |  |  | PD-MCI (n=32) | -1.598 | 1.072 | -4.500 | 1.303 | 0.840 |
|  | **Stroop false answers** | HC (n=26) | PD-NC (n=25) | -1.797 | 2.556 | -8.717 | 5.123 | 1.000 |
|  |  |  | PD-MCI (n=32) | -5.550 | 2.526 | -12.388 | 1.288 | 0.186 |
|  |  |  | PDD (n=31) | -19.206 | 3.174 | -27.799 | -10.613 | ***< 0.001**** |
|  |  | PD-NC (n=25) | HC (n=26) | 1.797 | 2.556 | -5.123 | 8.717 | 1.000 |
|  |  |  | PD-MCI (n=32) | -3.752 | 2.631 | -10.877 | 3.371 | 0.947 |
|  |  |  | PDD (n=31) | -17.409 | 3.258 | -26.231 | -8.587 | ***< 0.001**** |
|  |  | PD-MCI (n=32) | HC (n=26) | 5.550 | 2.526 | -1.288 | 12.388 | 0.186 |
|  |  |  | PD-NC (n=25) | 3.752 | 2.631 | -3.371 | 10.877 | 0.947 |
|  |  |  | PDD (n=31) | -13.656 | 3.234 | -22.413 | -4.898 | ***< 0.001**** |
|  |  | PDD (n=31) | HC (n=26) | 19.206 | 3.174 | 10.613 | 27.799 | ***< 0.001**** |
|  |  |  | PD-NC (n=25) | 17.409 | 3.258 | 8.587 | 26.231 | ***< 0.001**** |
|  |  |  | PD-MCI (n=32) | 13.656 | 3.234 | 4.898 | 22.413 | ***< 0.001**** |
|  | **Stroop time difference** | HC (n=26) | PD-NC (n=25) | -1345.835 | 373.214 | -2355.873 | -335.798 | **0.003*** |
|  |  |  | PD-MCI (n=32) | -22.403 | 364.694 | -1009.382 | 964.574 | 1.000 |
|  |  |  | PDD (n=31) | -17.562 | 463.398 | -1271.664 | 1236.539 | 1.000 |
|  |  | PD-NC (n=25) | HC (n=26) | 1345.835 | 373.214 | 335.798 | 2355.873 | **0.003*** |
|  |  |  | PD-MCI (n=32) | 1323.431 | 380.275 | 294.286 | 2352.577 | **0.005*** |
|  |  |  | PDD (n=31) | 1328.272 | 475.757 | 40.722 | 2615.823 | **0.039*** |
|  |  | PD-MCI (n=32) | HC (n=26) | 22.403 | 364.694 | -964.574 | 1009.382 | 1.000 |
|  |  |  | PD-NC (n=25) | -1323.431 | 380.275 | -2352.577 | -294.286 | **0.005*** |
|  |  |  | PDD (n=31) | 4.840 | 469.103 | -1264.701 | 1274.382 | 1.000 |
|  |  | PDD (n=31) | HC (n=26) | 17.562 | 463.398 | -1236.539 | 1271.664 | 1.000 |
|  |  |  | PD-NC (n=25) | -1328.272 | 475.757 | -2615.823 | -40.722 | **0.039*** |
|  |  |  | PD-MCI (n=32) | -4.840 | 469.103 | -1274.382 | 1264.701 | 1.000 |
|  | **Clock Drawing** | HC (n=26) | PD-NC (n=25) | 1.560 | 0.352 | 0.612 | 2.508 | ***< 0.001**** |
|  |  |  | PD-MCI (n=32) | 0.786 | 0.337 | -0.121 | 1.694 | 0.130 |
|  |  |  | PDD (n=31) | 2.423 | 0.345 | 1.493 | 3.354 | ***< 0.001**** |
|  |  | PD-NC (n=25) | HC (n=26) | -1.560 | 0.352 | -2.508 | -0.612 | ***< 0.001**** |
|  |  |  | PD-MCI (n=32) | -0.773 | 0.337 | -1.681 | 0.134 | 0.144 |
|  |  |  | PDD (n=31) | 0.863 | 0.345 | -0.066 | 1.794 | 0.085 |
|  |  | PD-MCI (n=32) | HC (n=26) | -0.786 | 0.337 | -1.694 | 0.121 | 0.130 |
|  |  |  | PD-NC (n=25) | 0.773 | 0.337 | -0.134 | 1.681 | 0.144 |
|  |  |  | PDD (n=31) | 1.637 | 0.330 | 0.747 | 2.526 | ***< 0.001**** |
|  |  | PDD (n=31) | HC (n=26) | -2.423 | 0.345 | -3.354 | -1.493 | ***< 0.001**** |
|  |  |  | PD-NC (n=25) | -0.863 | 0.345 | -1.794 | 0.066 | 0.085 |
|  |  |  | PD-MCI (n=32) | -1.637 | 0.330 | -2.526 | -0.747 | ***< 0.001**** |
| **Memory** | **Visual immediate recall** | HC (n=26) | PD-NC (n=25) | 4.375 | 0.806 | 2.204 | 6.546 | ***< 0.001**** |
|  |  |  | PD-MCI (n=32) | 3.472 | 0.784 | 1.361 | 5.583 | ***< 0.001**** |
|  |  |  | PDD (n=31) | 7.335 | 0.806 | 5.164 | 9.506 | ***< 0.001**** |
|  |  | PD-NC (n=25) | HC (n=26) | -4.375 | 0.806 | -6.546 | -2.204 | ***< 0.001**** |
|  |  |  | PD-MCI (n=32) | -0.902 | 0.792 | -3.035 | 1.229 | 1.000 |
|  |  |  | PDD (n=31) | 2.960 | 0.814 | 0.767 | 5.152 | **0.003*** |
|  |  | PD-MCI (n=32) | HC (n=26) | -3.472 | 0.784 | -5.583 | -1.361 | **< 0.001*** |
|  |  |  | PD-NC (n=25) | 0.902 | 0.792 | -1.229 | 3.035 | 1.000 |
|  |  |  | PDD (n=31) | 3.862 | 0.792 | 1.730 | 5.995 | **< 0.001*** |
|  |  | PDD (n=31) | HC (n=26) | -7.335 | 0.806 | -9.506 | -5.164 | **< 0.001*** |
|  |  |  | PD-NC (n=25) | -2.960 | 0.814 | -5.152 | -0.767 | **0.003*** |
|  |  |  | PD-MCI (n=32) | -3.862 | 0.792 | -5.995 | -1.730 | **< 0.001*** |
|  | **Visual delayed recall** | HC (n=26) | PD-NC (n=25) | 3.412 | 0.847 | 1.130 | 5.694 | **0.001*** |
|  |  |  | PD-MCI (n=32) | 3.299 | 0.824 | 1.080 | 5.518 | **0.001*** |
|  |  |  | PDD (n=31) | 6.972 | 0.847 | 4.690 | 9.254 | **< 0.001*** |
|  |  | PD-NC (n=25) | HC (n=26) | -3.412 | 0.847 | -5.694 | -1.130 | **0.001*** |
|  |  |  | PD-MCI (n=32) | -0.112 | 0.832 | -2.354 | 2.129 | 1.000 |
|  |  |  | PDD (n=31) | 3.560 | 0.856 | 1.255 | 5.864 | **< 0.001*** |
|  |  | PD-MCI (n=32) | HC (n=26) | -3.299 | 0.824 | -5.518 | -1.080 | **0.001*** |
|  |  |  | PD-NC (n=25) | 0.112 | 0.832 | -2.129 | 2.354 | 1.000 |
|  |  |  | PDD (n=31) | 3.672 | 0.832 | 1.430 | 5.914 | **< 0.001*** |
|  |  | PDD (n=31) | HC (n=26) | -6.972 | 0.847 | -9.254 | -4.690 | **< 0.001*** |
|  |  |  | PD-NC (n=25) | -3.560 | 0.856 | -5.864 | -1.255 | **< 0.001*** |
|  |  |  | PD-MCI (n=32) | -3.672 | 0.832 | -5.914 | -1.430 | **< 0.001*** |
|  | **Visual recognition** | HC (n=26) | PD-NC (n=25) | 0.681 | 0.339 | -0.235 | 1.598 | 0.287 |
|  |  |  | PD-MCI (n=32) | 0.957 | 0.323 | 0.084 | 1.831 | **0.024*** |
|  |  |  | PDD (n=31) | 1.954 | 0.339 | 1.037 | 2.871 | **< 0.001*** |
|  |  | PD-NC (n=25) | HC (n=26) | -0.681 | 0.339 | -1.598 | 0.235 | 0.287 |
|  |  |  | PD-MCI (n=32) | 0.276 | 0.323 | -0.597 | 1.149 | 1.000 |
|  |  |  | PDD (n=31) | 1.272 | 0.339 | 0.355 | 2.189 | **0.002*** |
|  |  | PD-MCI (n=32) | HC (n=26) | -0.957 | 0.323 | -1.831 | -0.084 | **0.024*** |
|  |  |  | PD-NC (n=25) | -0.276 | 0.323 | -1.149 | 0.597 | 1.000 |
|  |  |  | PDD (n=31) | 0.996 | 0.323 | 0.123 | 1.870 | **0.017*** |
|  |  | PDD (n=31) | HC (n=26) | -1.954 | 0.339 | -2.871 | -1.037 | **< 0.001*** |
|  |  |  | PD-NC (n=25) | -1.272 | 0.339 | -2.189 | -0.355 | **0.002*** |
|  |  |  | PD-MCI (n=32) | -0.996 | 0.323 | -1.870 | -0.123 | **0.017*** |
|  | **Logical immediate recall** | HC (n=26) | PD-NC (n=25) | 2.860 | 1.448 | -1.060 | 6.780 | 0.311 |
|  |  |  | PD-MCI (n=32) | 1.519 | 1.425 | -2.339 | 5.378 | 1.000 |
|  |  |  | PDD (n=31) | 8.977 | 1.425 | 5.118 | 12.836 | **< 0.001*** |
|  |  | PD-NC (n=25) | HC (n=26) | -2.860 | 1.448 | -6.780 | 1.060 | 0.311 |
|  |  |  | PD-MCI (n=32) | -1.340 | 1.222 | -4.648 | 1.966 | 1.000 |
|  |  |  | PDD (n=31) | 6.117 | 1.222 | 2.809 | 9.425 | **< 0.001*** |
|  |  | PD-MCI (n=32) | HC (n=26) | -1.519 | 1.425 | -5.378 | 2.339 | 1.000 |
|  |  |  | PD-NC (n=25) | 1.340 | 1.222 | -1.966 | 4.648 | 1.000 |
|  |  |  | PDD (n=31) | 7.458 | 1.195 | 4.223 | 10.693 | **< 0.001*** |
|  |  | PDD (n=31) | HC (n=26) | -8.977 | 1.425 | -12.836 | -5.118 | **< 0.001*** |
|  |  |  | PD-NC (n=25) | -6.117 | 1.222 | -9.425 | -2.809 | **< 0.001*** |
|  |  |  | PD-MCI (n=32) | -7.458 | 1.195 | -10.693 | -4.223 | **< 0.001*** |
|  | **Logical delayed recall** | HC (n=26) | PD-NC (n=25) | 3.465 | 1.589 | -0.835 | 7.765 | 0.193 |
|  |  |  | PD-MCI (n=32) | 3.358 | 1.564 | -0.874 | 7.592 | 0.209 |
|  |  |  | PDD (n=31) | 11.192 | 1.564 | 6.959 | 15.425 | **< 0.001*** |
|  |  | PD-NC (n=25) | HC (n=26) | -3.465 | 1.589 | -7.765 | 0.835 | 0.193 |
|  |  |  | PD-MCI (n=32) | -0.106 | 1.340 | -3.734 | 3.522 | 1.000 |
|  |  |  | PDD (n=31) | 7.727 | 1.340 | 4.098 | 11.355 | **< 0.001*** |
|  |  | PD-MCI (n=32) | HC (n=26) | -3.358 | 1.564 | -7.592 | 0.874 | 0.209 |
|  |  |  | PD-NC (n=25) | 0.106 | 1.340 | -3.522 | 3.734 | 1.000 |
|  |  |  | PDD (n=31) | 7.833 | 1.311 | 4.284 | 11.382 | **< 0.001*** |
|  |  | PDD (n=31) | HC (n=26) | -11.192 | 1.564 | -15.425 | -6.959 | **< 0.001*** |
|  |  |  | PD-NC (n=25) | -7.727 | 1.340 | -11.355 | -4.098 | **< 0.001*** |
|  |  |  | PD-MCI (n=32) | -7.833 | 1.311 | -11.382 | -4.284 | **< 0.001*** |
|  | **VMPT immediate recall** | HC (n=26) | PD-NC (n=25) | 1.826 | 0.475 | 0.547 | 3.106 | **0.001*** |
|  |  |  | PD-MCI (n=32) | 2.442 | 0.434 | 1.273 | 3.612 | **< 0.001*** |
|  |  |  | PDD (n=31) | 3.529 | 0.443 | 2.334 | 4.724 | **< 0.001*** |
|  |  | PD-NC (n=25) | HC (n=26) | -1.826 | 0.475 | -3.106 | -0.547 | **0.001*** |
|  |  |  | PD-MCI (n=32) | 0.615 | 0.440 | -0.569 | 1.801 | 0.990 |
|  |  |  | PDD (n=31) | 1.702 | 0.449 | 0.491 | 2.913 | 0.002 |
|  |  | PD-MCI (n=32) | HC (n=26) | -2.442 | 0.434 | -3.612 | -1.273 | **< 0.001*** |
|  |  |  | PD-NC (n=25) | -0.615 | 0.440 | -1.801 | 0.569 | 0.990 |
|  |  |  | PDD (n=31) | 1.086 | 0.406 | -0.007 | 2.180 | 0.053 |
|  |  | PDD (n=31) | HC (n=26) | -3.529 | 0.443 | -4.724 | -2.334 | **< 0.001*** |
|  |  |  | PD-NC (n=25) | -1.702 | 0.449 | -2.913 | -0.491 | **0.002*** |
|  |  |  | PD-MCI (n=32) | -1.086 | 0.406 | -2.180 | 0.007 | 0.053 |
|  | **VMPT delayed recall** | HC (n=26) | PD-NC (n=25) | 5.301 | 0.825 | 3.082 | 7.520 | **< 0.001*** |
|  |  |  | PD-MCI (n=32) | 4.947 | 0.770 | 2.877 | 7.016 | **< 0.001*** |
|  |  |  | PDD (n=31) | 7.836 | 0.775 | 5.751 | 9.920 | **< 0.001*** |
|  |  | PD-NC (n=25) | HC (n=26) | -5.301 | 0.825 | -7.520 | -3.082 | **< 0.001*** |
|  |  |  | PD-MCI (n=32) | -0.354 | 0.787 | -2.470 | 1.762 | 1.000 |
|  |  |  | PDD (n=31) | 2.534 | 0.793 | 0.403 | 4.666 | **0.011*** |
|  |  | PD-MCI (n=32) | HC (n=26) | -4.947 | 0.770 | -7.016 | -2.877 | **< 0.001*** |
|  |  |  | PD-NC (n=25) | 0.354 | 0.787 | -1.762 | 2.470 | 1.000 |
|  |  |  | PDD (n=31) | 2.889 | 0.735 | 0.913 | 4.864 | **0.001*** |
|  |  | PDD (n=31) | HC (n=26) | -7.836 | 0.775 | -9.920 | -5.751 | **< 0.001*** |
|  |  |  | PD-NC (n=25) | -2.534 | 0.793 | -4.666 | -0.403 | **0.011*** |
|  |  |  | PD-MCI (n=32) | -2.889 | 0.735 | -4.864 | -0.913 | **0.001*** |
|  | **VMPT recognition** | HC (n=26) | PD-NC (n=25) | -0.490 | 0.780 | -2.588 | 1.607 | 1.000 |
|  |  |  | PD-MCI (n=32) | -3.177 | 0.728 | -5.134 | -1.221 | **< 0.001*** |
|  |  |  | PDD (n=31) | -0.905 | 0.733 | -2.876 | 1.065 | 1.000 |
|  |  | PD-NC (n=25) | HC (n=26) | 0.490 | 0.780 | -1.607 | 2.588 | 1.000 |
|  |  |  | PD-MCI (n=32) | -2.687 | 0.744 | -4.688 | -0.686 | **0.003*** |
|  |  |  | PDD (n=31) | -0.415 | 0.749 | -2.430 | 1.599 | 1.000 |
|  |  | PD-MCI (n=32) | HC (n=26) | 3.177 | 0.728 | 1.221 | 5.134 | **< 0.001*** |
|  |  |  | PD-NC (n=25) | 2.687 | 0.744 | 0.686 | 4.688 | **0.003*** |
|  |  |  | PDD (n=31) | 2.272 | 0.695 | 0.404 | 4.139 | **0.009*** |
|  |  | PDD (n=31) | HC (n=26) | 0.905 | 0.733 | -1.065 | 2.876 | 1.000 |
|  |  |  | PD-NC (n=25) | 0.415 | 0.749 | -1.599 | 2.430 | 1.000 |
|  |  |  | PD-MCI (n=32) | -2.272 | 0.695 | -4.139 | -0.404 | **0.009*** |
|  | **VMPT Total** | HC (n=26) | PD-NC (n=25) | 3.113 | 1.009 | 0.385 | 5.841 | **0.017*** |
|  |  |  | PD-MCI (n=32) | 1.670 | 0.681 | -0.172 | 3.512 | 0.098 |
|  |  |  | PDD (n=31) | 6.577 | 0.696 | 4.695 | 8.460 | **< 0.001*** |
|  |  | PD-NC (n=25) | HC (n=26) | -3.113 | 1.009 | -5.841 | -0.385 | **0.017*** |
|  |  |  | PD-MCI (n=32) | -1.443 | 0.970 | -4.064 | 1.177 | 0.843 |
|  |  |  | PDD (n=31) | 3.464 | 0.980 | 0.815 | 6.113 | **0.004*** |
|  |  | PD-MCI (n=32) | HC (n=26) | -1.670 | 0.681 | -3.512 | 0.172 | 0.098 |
|  |  |  | PD-NC (n=25) | 1.443 | 0.970 | -1.177 | 4.064 | 0.843 |
|  |  |  | PDD (n=31) | 4.907 | 0.637 | 3.185 | 6.630 | **< 0.001*** |
|  |  | PDD (n=31) | HC (n=26) | -6.577 | 0.696 | -8.460 | -4.695 | **< 0.001*** |
|  |  |  | PD-NC (n=25) | -3.464 | 0.980 | -6.113 | -0.815 | **0.004*** |
|  |  |  | PD-MCI (n=32) | -4.907 | 0.637 | -6.630 | -3.185 | **< 0.001*** |
| **Language** | **Boston Naming Test** | HC (n=26) | PD-NC (n=25) | 4.320 | 1.968 | -0.981 | 9.622 | 0.183 |
|  |  |  | PD-MCI (n=32) | 5.434 | 1.823 | 0.522 | 10.346 | **0.022*** |
|  |  |  | PDD (n=31) | 9.586 | 1.892 | 4.487 | 14.685 | **< 0.001*** |
|  |  | PD-NC (n=25) | HC (n=26) | -4.320 | 1.968 | -9.622 | 0.981 | 0.183 |
|  |  |  | PD-MCI (n=32) | 1.114 | 1.798 | -3.730 | 5.959 | 1.000 |
|  |  |  | PDD (n=31) | 5.265 | 1.868 | 0.231 | 10.300 | **0.035*** |
|  |  | PD-MCI (n=32) | HC (n=26) | -5.434 | 1.823 | -10.346 | -0.522 | **0.022*** |
|  |  |  | PD-NC (n=25) | -1.114 | 1.798 | -5.959 | 3.730 | 1.000 |
|  |  |  | PDD (n=31) | 4.151 | 1.715 | -0.470 | 8.773 | 0.104 |
|  |  | PDD (n=31) | HC (n=26) | -9.586 | 1.892 | -14.685 | -4.487 | **< 0.001*** |
|  |  |  | PD-NC (n=25) | -5.265 | 1.868 | -10.300 | -0.231 | **0.035*** |
|  |  |  | PD-MCI (n=32) | -4.151 | 1.715 | -8.773 | 0.470 | 0.104 |
| **Visual Spatial Functions** | **Benton Face Recognition** | HC (n=26) | PD-NC (n=25) | 1.140 | 1.889 | -3.939 | 6.219 | 1.000 |
|  |  |  | PD-MCI (n=32) | 5.281 | 1.785 | 0.481 | 10.080 | **0.023*** |
|  |  |  | PDD (n=31) | 6.725 | 1.797 | 1.893 | 11.558 | **0.002*** |
|  |  | PD-NC (n=25) | HC (n=26) | -1.140 | 1.889 | -6.219 | 3.939 | 1.000 |
|  |  |  | PD-MCI (n=32) | 4.141 | 1.765 | -0.602 | 8.885 | 0.125 |
|  |  |  | PDD (n=31) | 5.585 | 1.777 | 0.808 | 10.363 | **0.013*** |
|  |  | PD-MCI (n=32) | HC (n=26) | -5.281 | 1.785 | -10.080 | -0.481 | **0.023*** |
|  |  |  | PD-NC (n=25) | -4.141 | 1.765 | -8.885 | 0.602 | 0.125 |
|  |  |  | PDD (n=31) | 1.444 | 1.666 | -3.034 | 5.923 | 1.000 |
|  |  | PDD (n=31) | HC (n=26) | -6.725 | 1.797 | -11.558 | -1.893 | **0.002*** |
|  |  |  | PD-NC (n=25) | -5.585 | 1.777 | -10.363 | -0.808 | **0.013*** |
|  |  |  | PD-MCI (n=32) | -1.444 | 1.666 | -5.923 | 3.034 | 1.000 |
|  | **Line Orientation** | HC (n=26) | PD-NC (n=25) | -14.250 | 2.907 | -22.088 | -6.411 | **< 0.001*** |
|  |  |  | PD-MCI (n=32) | 11.125 | 2.719 | 3.793 | 18.457 | **0.001*** |
|  |  |  | PDD (n=31) | 17.272 | 3.192 | 8.664 | 25.879 | **< 0.001*** |
|  |  | PD-NC (n=25) | HC (n=26) | 14.250 | 2.907 | 6.411 | 22.0882 | **< 0.001*** |
|  |  |  | PD-MCI (n=32) | 25.375 | 2.719 | 18.043 | 32.707 | **< 0.001*** |
|  |  |  | PDD (n=31) | 31.522 | 3.192 | 22.914 | 40.129 | **< 0.001*** |
|  |  | PD-MCI (n=32) | HC (n=26) | -11.125 | 2.719 | -18.457 | -3.793 | **0.001*** |
|  |  |  | PD-NC (n=25) | -25.375 | 2.719 | -32.707 | -18.043 | **< 0.001*** |
|  |  |  | PDD (n=31) | 6.147 | 3.022 | -2.002 | 14.296 | 0.269 |
|  |  | PDD (n=31) | HC (n=26) | -17.272 | 3.192 | -25.879 | -8.664 | **< 0.001*** |
|  |  |  | PD-NC (n=25) | -31.522 | 3.192 | -40.129 | -22.914 | **< 0.001*** |
|  |  |  | PD-MCI (n=32) | -6.147 | 3.022 | -14.296 | 2.002 | 0.269 |
| **General cognition** | **MMSE** | HC (n=26) | PD-NC (n=25) | 1.415 | 0.796 | -0.724 | 3.555 | 0.470 |
|  |  |  | PD-MCI (n=32) | 3.959 | 0.750 | 1.942 | 5.976 | **< 0.001*** |
|  |  |  | PDD (n=31) | 8.550 | 0.756 | 6.519 | 10.582 | **< 0.001*** |
|  |  | PD-NC (n=25) | HC (n=26) | -1.415 | 0.796 | -3.555 | 0.724 | 0.470 |
|  |  |  | PD-MCI (n=32) | 2.543 | 0.758 | 0.504 | 4.582 | **0.007*** |
|  |  |  | PDD (n=31) | 7.135 | 0.764 | 5.082 | 9.189 | **< 0.001*** |
|  |  | PD-MCI (n=32) | HC (n=26) | -3.959 | 0.750 | -5.976 | -1.942 | **< 0.001*** |
|  |  |  | PD-NC (n=25) | -2.543 | 0.758 | -4.582 | -0.504 | **0.007*** |
|  |  |  | PDD (n=31) | 4.591 | 0.716 | 2.666 | 6.516 | **< 0.001*** |
|  |  | PDD (n=31) | HC (n=26) | -8.550 | 0.756 | -10.582 | -6.519 | **< 0.001*** |
|  |  |  | PD-NC (n=25) | -7.135 | 0.764 | -9.189 | -5.082 | **< 0.001*** |
|  |  |  | PD-MCI (n=32) | -4.591 | 0.716 | -6.516 | -2.666 | **< 0.001*** |

PD: Parkinson’s Disease; HC: Healthy Control; PD-NC: PD-Normal Cognition; PD-MCI: PD-Mild Cognitive Impairment; PDD: PD Dementia, VMPT: Verbal Memory Process Test, MMSE: Mini-Mental Standard Examination Test. *p<0.05
