# Supplementary figures and images for: Functional neural networks stratify Parkinson's disease patients across the spectrum of cognitive impairment
Source: Brain Behav. 2024 Jan 24;14(1):e3395. doi: 10.1002/brb3.3395 (PMC10808882; doi:10.1002/brb3.3395)

# HC

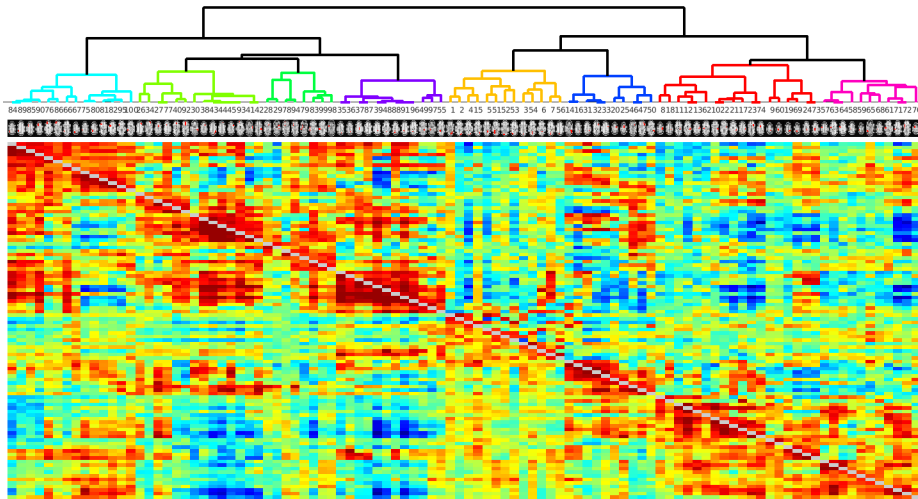

# PD-NC

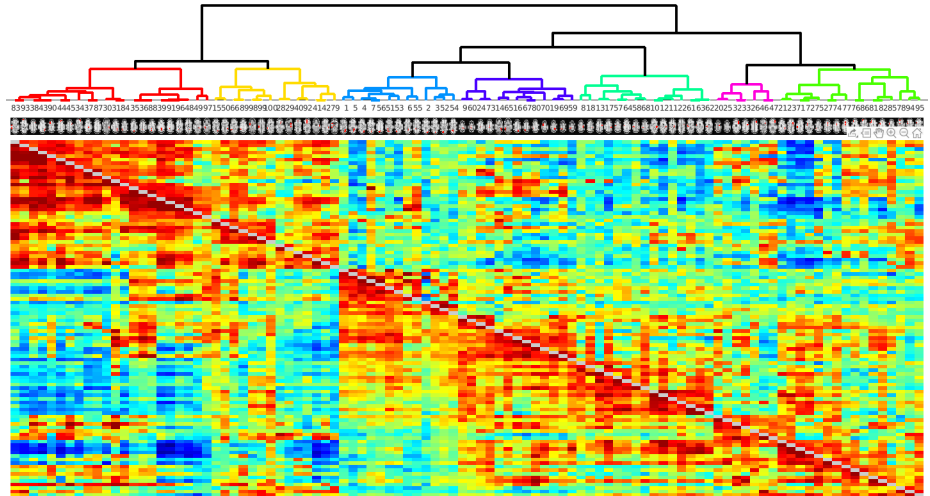

# PD-MCI

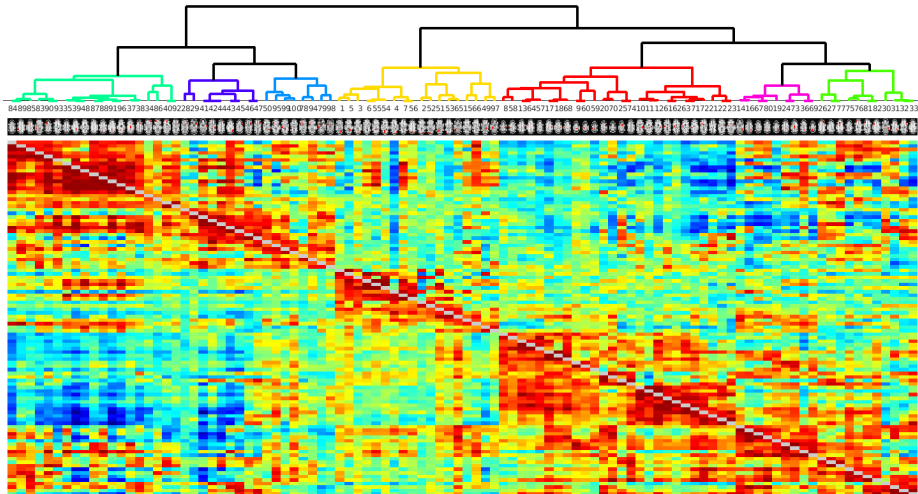

# PDD

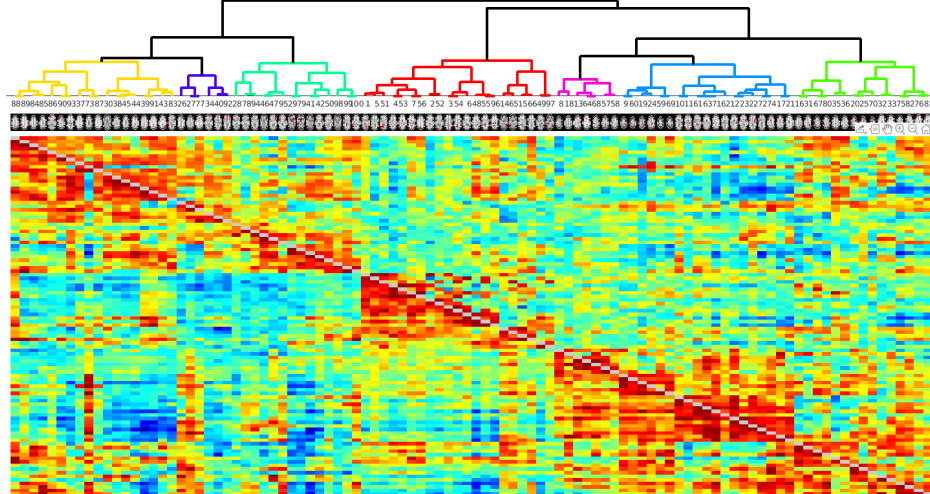

Supplement: Supplementary file 2 — Hierarchical clustering dendrogram in four groups (see “Methods”, inter‐network connectivity analysis). Coloring in the dendrogram indicates main clusters with high temporal correlations thresholded at 0.75, as a default value in nets_hierarchy of FLSNets. The upper and lower triangular matrices represent the partial and full correlations in all the dendrograms. [file BRB3-14-e3395-s001.pdf]
